# The Lives Saved Tool (*LiST*) as a model for diarrhea mortality reduction

**DOI:** 10.1186/1741-7015-12-70

**Published:** 2014-04-29

**Authors:** Christa L Fischer Walker, Neff Walker

**Affiliations:** 1 Department of International Health Johns Hopkins Bloomberg School of Public Health, Institute for International Programs, Baltimore, MD, USA

**Keywords:** Diarrhea, Modeling, Enteric diseases, Child health, Maternal health

## Abstract

**Background:**

Diarrhea is a leading cause of morbidity and mortality among children under five years of age. The Lives Saved Tool (*LiST*) is a model used to calculate deaths averted or lives saved by past interventions and for the purposes of program planning when costly and time consuming impact studies are not possible.

**Discussion:**

*LiST* models the relationship between coverage of interventions and outputs, such as stunting, diarrhea incidence and diarrhea mortality. Each intervention directly prevents a proportion of diarrhea deaths such that the effect size of the intervention is multiplied by coverage to calculate lives saved. That is, the maximum effect size could be achieved at 100% coverage, but at 50% coverage only 50% of possible deaths are prevented. Diarrhea mortality is one of the most complex causes of death to be modeled*.* The complexity is driven by the combination of direct prevention and treatment interventions as well as interventions that operate indirectly via the reduction in risk factors, such as stunting and wasting. Published evidence is used to quantify the effect sizes for each direct and indirect relationship. Several studies have compared measured changes in mortality to *LiST* estimates of mortality change looking at different sets of interventions in different countries. While comparison work has generally found good agreement between the *LiST* estimates and measured mortality reduction, where data availability is weak, the model is less likely to produce accurate results. *LiST* can be used as a component of program evaluation, but should be coupled with more complete information on inputs, processes and outputs, not just outcomes and impact.

**Summary:**

*LiST* is an effective tool for modeling diarrhea mortality and can be a useful alternative to large and expensive mortality impact studies. Predicting the impact of interventions or comparing the impact of more than one intervention without having to wait for the results of large and expensive mortality studies is critical to keep programs focused and results oriented for continued reductions in diarrhea and all-cause mortality among children under five years of age.

## Background

Diarrhea is a leading cause of morbidity and mortality among children under five years of age around the world [1]. Preventing and treating infectious diarrhea does not require expensive or technologically complex interventions; rather, with high coverage of seven simple interventions the burden of diarrhea could be greatly reduced [2]. Prevention interventions include regular hand washing with soap, improved access to clean water and sanitation facilities, breastfeeding, routine vitamin A supplementation and rotavirus vaccination. New diarrhea treatment guidelines came out in 2004, introducing zinc supplementation and low osmolarity oral rehydration salts (ORS) for the treatment of all diarrhea episodes [3]. Evaluating these diarrhea management interventions within a broad child health framework is critical if we are to continue to understand where new programs are achieving success and where some have failed to reach young children.

A rigorous prospective program evaluation includes components of inputs, processes, outputs, outcomes and impact [4], but often collecting quality data at each of these levels while also collecting critical information with regard to contextual variables is not fiscally feasible. Impact measures, such as reduction in diarrhea specific mortality or stunting rates, are the most difficult to measure because sample sizes for reduction in cause-specific mortality are extremely large, even if it is anticipated that the targeted program achieved a successful scale up. The Lives Saved Tool (*LiST*) is a model used to calculate deaths averted or lives saved by past interventions and for the purposes of program planning when costly and time consuming impact studies are not possible. In this paper we will review the components of *LiST* that are critical for modeling diarrhea mortality. We will explain how the model includes direct and indirect effects on diarrhea mortality, sources for current *LiST* estimates, and discuss the benefits and weaknesses of using *LiST* as an alternative to measured diarrhea mortality. This is the first paper to describe the full *LiST* model with regard to the complexity of diarrheal disease.

## Discussion

### Lives saved tool (*LiST):* background and history

The ***Lives Saved Tool *(*LiST*)** has been developed over the past 10 years. The initial version of the software was developed as part of the work for the Child Survival Series published in *Lancet* in 2003 [5,6]. The purpose of the program was to estimate the impact that scaling up community-based interventions would have on under-five mortality [7], with both a limited set of interventions and outputs and very limited demographic capability. Starting from this initial point the software was expanded first to handle a new set of interventions that focused more on facility-based care with the primary impact being on neonatal mortality [8,9]. The model was then improved to handle populations and cohorts and to include wasting and stunting as risk factors as part of the work for the *Lancet* Nutrition Series [10]. This further development of the software was supported by the Bill & Melinda Gates Foundation as part of the work of the Child Health and Epidemiology Reference Group (CHERG). The software was then shifted into the free and publicly available Spectrum software package, to take advantage of the demographic capabilities in that software and to provide links to the AIDS Impact Model module that had been developed to estimate the impact of HIV/AIDs [11]. Since that time, *LiST* has expanded its scope to look at the impact of interventions on birth outcomes and stillbirths [12], maternal mortality, and incidence of pneumonia and diarrhea [13] as well as neonatal and child mortality.

### Theoretical approach and basic modeling structure of *LiST*

*LiST* is characterized as a linear, mathematical model that is deterministic [14]. It describes fixed relationships between inputs and outputs that will produce the same outputs each time one runs the model. In *LiST* the primary inputs are coverage of interventions and the outputs are changes in population level of risk factors (such as wasting or stunting rates, birth outcomes, such as prematurity or size at birth) and cause-specific mortality (neonatal, child mortality 1 to 59 months, maternal mortality and stillbirths). The relationship between an input (change in intervention coverage) with one or more outputs is specified in terms of the effectiveness of the intervention in reducing the probability of that outcome. The outcome can be cause-specific mortality or a risk factor. The primary underlying assumption in *LiST* is that mortality rates and cause of death structure will not change except in response to changes in coverage of interventions. The assumption is that changes in distal variables, such as increase in per capita income or mothers’ education, will decrease cause-specific mortality, such as diarrhea mortality, by increasing coverage of interventions or reducing risk factors, such as increased rates of exclusive breastfeeding.

Currently there are around 67 interventions in *LiST,* although the number of interventions that are in *LiST* is growing as new interventions are added. These interventions have an impact on stillbirths, neonatal mortality, mortality in children 1 to 59 months, maternal mortality or risk factors within the model. Interventions can be linked to multiple causes of death and risk factors. A key feature of *LiST* is that it allows one to look at the impact of scaling up coverage of multiple interventions simultaneously, instead of a single intervention and one cause as is done in many natural history models. As an example, this has been done for the scale up of prevention and treatment interventions for diarrhea mortality [15]. Authors demonstrated that diarrhea mortality could be reduced by at least 78% with ambitious, yet feasible, improvements in the coverage of all key diarrhea prevention and treatment interventions by 2015 in 68 priority low- and middle-income countries.

There are structural features about the model that must be considered in order to estimate the impact of scaling up coverage of multiple diarrhea interventions and changes in risk factors on diarrhea mortality. First, the effectiveness or efficacy of an intervention must be described in terms of reduction in cause-specific mortality rather than in overall mortality. For example, the effect size of vitamin A supplementation is usually cited as it relates to a reduction in all-cause mortality, but *LiST* requires a reduction in all-cause mortality be converted to a reduction in cause-specific mortality. For Vitamin A, this means that the reduction in mortality must be attributable to one or more causes of death, such as diarrhea deaths, and each requires an accompanying effect size. With cause-specific estimates of efficacy we can then compute the combined impact of interventions.

Within *LiST,* efficacy of an intervention is defined in terms of the reduction of a cause of death or risk factor. When there is a single intervention the calculation of impact is simple as one has change in coverage times the efficacy of the intervention and this is applied to the cause specific mortality. For example, if in country X we have 10,000 diarrhea deaths among children aged 1 to 59 months/year and we introduce a new vaccine that is 50% effective in reducing diarrhea mortality we can estimate the number of lives we would save. If coverage were to reach 50% of children we would then reduce diarrhea mortality to 7,500 (10,000 – (10,000 *0.5 * 0.5)) diarrhea deaths/year. When there is a second or a third intervention, the same approach is followed except that the second diarrhea intervention would be applied to the residual diarrhea deaths. So, if a second new diarrhea intervention is also 50% effective and coverage reaches 50% we would further reduce diarrhea mortality to 5,626 deaths/year. By using cause-specific efficacy and applying each intervention to the residual deaths after the previous intervention we ensure that we are not double counting the impact of interventions. Within *LiST* users are able to include any number of prevention and treatment interventions to model real changes in coverage or to create new scenarios. Any time multiple interventions are being modeled at one time, the preventive interventions will act on diarrhea mortality first. Treatment interventions will then prevent the residual of deaths as was described above. When a user is interested in a total number of deaths prevented by a package of interventions, this differentiation occurs in the model (that is, behind the scenes) and is not important because deaths are not attributable to one specific intervention in the output. However, if a user is interested in intervention specific deaths prevented, it may be necessary to run *LiST* several times isolating individual interventions in addition to a package of interventions to understand more fully the potential contribution of unique interventions.

### Modeling reduction in diarrhea mortality using *LiST*

**Figure 1:**
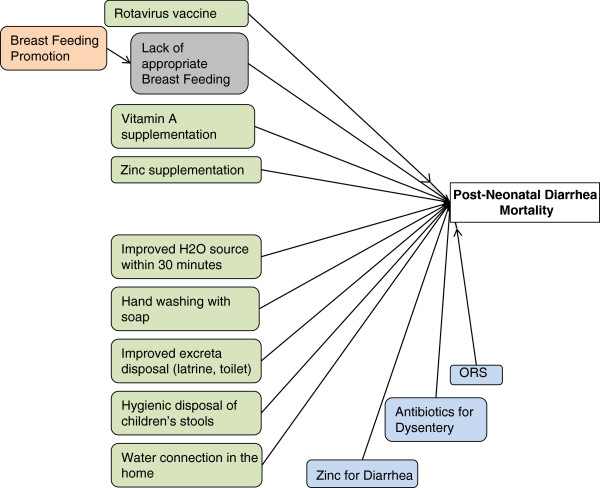
**Interventions with direct impact on diarrhea mortality.** Green shaded boxes indicate prevention interventions. Blue shaded boxes indicate treatment interventions. Peach and grey boxes indicate interventions that have an impact on diarrhea via a risk factor pathway.

Diarrhea mortality is one of the most complex causes of death to be modeled within *LiST.* The complexity is driven by the combination of direct prevention and treatment interventions as well as interventions that operate indirectly via the reduction in risk factors, such as stunting and wasting. Figure 1 illustrates the interventions that have direct impacts on diarrhea mortality within *LiST.* For prevention, they include: rotavirus vaccine; vitamin A supplementation for children 6 to 59 months of age every six months; preventive zinc supplementation; age appropriate breastfeeding; and water, sanitation, and hygiene interventions at the individual and household levels.

Each of these interventions directly prevents a proportion of diarrhea deaths. For example, the effectiveness estimate for zinc for the treatment of diarrhea is 23%. In *LiST* to achieve a reduction in mortality, the effect size is multiplied by the coverage and assumes that all other interventions are kept constant. Thus, at 100% coverage, zinc treatment could prevent 23% of current diarrhea deaths among children 1 to 59 months of age. If coverage were halved to 50%, the deaths prevented would also be reduced by 50%. The sources of data for the generation of effect sizes for all interventions are discussed below and all point estimates are provided in a detailed Additional file 1. Appropriate breastfeeding is slightly different than the aforementioned preventive interventions included in *LiST.* Breastfeeding is included in two parts, the action of breastfeeding and the intervention of breastfeeding promotion. With regard to mortality, the lack of appropriate breastfeeding can be modeled as a risk factor. In *LiST*, the proportion of children receiving appropriate breastfeeding can be directly changed over time (to see the impact of changing breastfeeding rates) or it can be modeled as a risk factor that changes with the intervention of breastfeeding promotion shifting rates of breastfeeding practices.

There are three interventions for the treatment of diarrhea: zinc treatment, ORS, and antibiotics for dysentery that directly impact diarrhea mortality in the model. One important concept in *LiST* is that some interventions are able to act on diarrhea deaths that are caused by a specific etiological agent or agents. This is the case of antibiotics for the treatment of dysentery. In *LiST,* antibiotics are only able to prevent dysentery deaths and, thus, only prevent that proportion of all diarrhea deaths. This is termed the ‘affected fraction’ in *LiST.* Details of each intervention are explained in subsequent sections.

**Figure 2:**
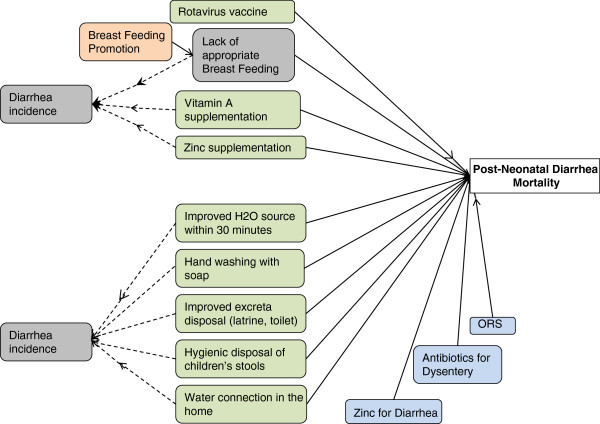
**Interventions impact direct mortality and diarrhea incidence.** Green shaded boxes indicate prevention interventions. Blue shaded boxes indicate treatment interventions. Peach and grey boxes indicate interventions that have an impact on diarrhea via a risk factor pathway.

Most causes of death in *LiST* do not include disease incidence in the pathway because the primary purpose of *LiST* is to model mortality reductions. However, diarrhea incidence is critical in the stunting/mortality pathway so it is included in the *LiST* tool. Figure 2 illustrates the interventions that impact diarrhea incidence. Given that diarrhea incidence is in *LiST* in addition to diarrhea mortality, a user could model changes in diarrhea incidence as a result of the scale up of diarrhea prevention and treatment interventions, but this is rarely of interest when using *LiST.*

**Figure 3:**
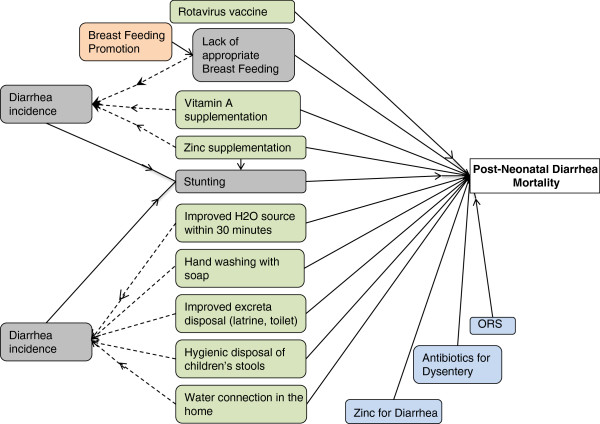
**Interventions impact diarrhea directly and via the stunting pathway.** Green shaded boxes indicate prevention interventions. Blue shaded boxes indicate treatment interventions. Peach and grey boxes indicate interventions that have an impact on diarrhea via a risk factor pathway.

Figure 3 illustrates how the preventive interventions that decrease diarrhea incidence, decrease stunting, which then decreases overall diarrhea mortality during the post neonatal period. For these interventions, *LiST* includes values for the effectiveness of the intervention on direct mortality reduction and the reduction on diarrhea incidence. The impact of diarrhea incidence on stunting is then calculated based on data from a pooled analysis of several large cohort datasets [16]. Finally, the risk of stunting on diarrhea mortality is modeled using data from cohort and interventions studies that observed an increased risk of diarrhea mortality as the degree of stunting increases [17].

**Figure 4:**
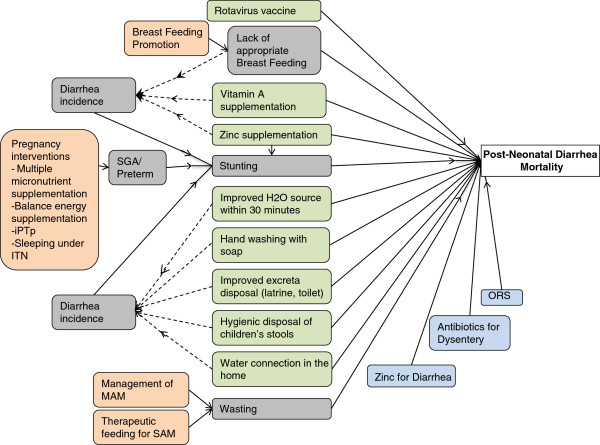
**Complete set of interventions impacting diarrhea mortality.** Green shaded boxes indicate preventive interventions. Blue shaded boxes indicate treatment interventions. Peach and grey boxes indicate interventions via a risk factor pathway.

Lastly, there are several interventions that can be delivered during pregnancy that reduce the risk of preterm births and/or the risk of an infant being small for gestational age (SGA). Both preterm delivery and SGA are risk factors for stunting and are thus included in the complete set of interventions impacting diarrhea mortality in Figure 4. Wasting is also a risk factor for diarrhea mortality and thus interventions that reduce the prevalence of wasting will also contribute to a reduction in diarrhea mortality. This last group of interventions is not typically thought of as diarrhea-specific prevention and treatment interventions and further discussion of these interventions is beyond the scope of this report. They are included in Additional file 1.

### Interventions directly impacting diarrhea mortality in *LiST*

#### Impact of breastfeeding on diarrhea mortality

Breastfeeding is the best source of nutrition for young infants and an important tool in the prevention of diarrhea morbidity and mortality. The current WHO recommendations are for exclusive breastfeeding from birth until six month of age and continued breastfeeding until two years of age [18]. The *LiST* tool includes breastfeeding as a critical tool for the prevention of diarrhea morbidity and mortality, quantifying the increased risk of suboptimal feeding from 0 to 5 months, 6 to 11 months and 12 to 23 months of age. The effect sizes included in the *LiST* tool are presented in Additional file 1. To generate these estimates, Lamberti and colleagues conducted a systematic literature review to identify intervention and observational cohort studies. Few prospective studies are designed and powered to detect differences in diarrhea mortality so the authors included outcomes of incidence and severe disease to provide additional evidence. Assessing the effect size across disease severity is beneficial because it allows for a more complete picture of the evidence even if all estimates are not included in the final *LiST* tool. There are two pathways by which breastfeeding can decrease the risk of mortality in the *LiST* model. The first is by directly lowering the risk of direct mortality, which is likely due to a reduction in the risk of having a severe and/or prolonged episode of diarrhea. The second is by decreasing diarrhea incidence, which has an indirect effect on diarrhea mortality.

When reviewing the evidence of association between lack of breastfeeding and risk of disease or mortality it is important to minimize the bias of reverse causality. This occurs when the mother stops breastfeeding or introduces other liquids or foods to the child in response to an illness, such as diarrhea. This bias can be minimized by including only prospective studies in the generation of effect sizes as was done in Lamberti *et al*. [18].

#### Impact of breastfeeding promotion on breastfeeding patterns

Community or hospital-based interventions, such as education and counseling, are needed to increase breastfeeding rates. *LiST* allows users to evaluate the impact of changing breastfeeding patterns on mortality in two ways, through increasing the coverage of breastfeeding promotion or by directly changing the breastfeeding patterns in four age periods. For *LiST*, there have been reviews that estimated the impact of breastfeeding promotion on adoption of correct breastfeeding patterns [19,20]. The final effect size generated by these reviews included all studies regardless of study quality and did not attempt to adjust for or categorize the results according to the intensity of the intervention delivered. For a user looking to measure the effect of breastfeeding promotion on diarrhea morbidity and/or mortality, additional knowledge about the setting, dose and measured impact on breastfeeding rates may improve the estimate.

The estimated relative risk of appropriate breastfeeding given breastfeeding promotion is used to estimate the change in appropriate breastfeeding in each of the periods. For the time periods 0 to 1 month and 1 to 5 months the optimal breastfeeding is exclusive breastfeeding and for the periods of 6 to 11 months and 12 to 23 months the optimal breastfeeding is any/continued breastfeeding. For the period under six months, the remaining sub-optimal breastfeeding is distributed among the three sub-optimal categories (predominate, partial and none) based on their proportions prior to breastfeeding promotion scale up. An example of how breastfeeding promotion can change the proportion of babies receiving different degrees of breastfeeding is provided in Table 1.

**Table 1:** Illustration of the effect of breastfeeding promotion on the proportion of infants breastfed, by degree of breastfeeding and age

	**0 to 1 month**		**1 to 5 months**	
**Breastfeeding**	**Baseline**	**After promotion**	**Breastfeeding**	**Baseline**
**Exclusive**	62.0%	69.6%	40.2%	54.5%
**Predominant**	25.0%	20.0%	29.1%	22.1%
**Partial**	10.3%	8.2%	29.2%	22.2%
**None**	2.7%	2.2%	1.5%	1.1%

Breastfeeding promotion scaled from 40% to 80% of pregnant women.

In addition to using breastfeeding promotion to alter breastfeeding patterns, users can also directly change the breastfeeding rates. This allows users to estimate the optimal impact of improving breastfeeding as well as allowing users to look at historical patterns to estimate the impact of changes in breastfeeding on under-five mortality.

#### Impact of rotavirus vaccine on diarrhea mortality

Rotavirus is the leading cause of diarrhea mortality among children under five years of age [21]. In 2009, data from new studies in Asia and Africa were released and the World Health Organization began recommending rotavirus vaccine for routine vaccination of infants in all countries [22]. The current estimate used in the *LiST* model includes a combined effect size for the two oral, live, attenuated vaccines on the market and available as of January 2009, Rotarix (GlaxoSmithKline) and RotaTeq (Merck & Co, Inc.) [23]. The most recent review of the evidence included data from four new studies in Asia and sub-Saharan Africa. The *LiST* model includes regional specific effect sizes. The original rotavirus vaccine trials did not measure diarrhea mortality; the most severe outcome has been severe rotavirus morbidity, which is thus used as a proxy for mortality in the model [23]. In addition, rotavirus vaccine reduces rotavirus diarrhea incidence by 50% [24].

The proportion of diarrhea deaths attributable to rotavirus differs by setting. Ideally, these measures would be available at the country level, but this is rarely the case. As more data become available at the regional or country level, these details can be fed into *LiST* and continuous updates generated. For now, *LiST* uses regional estimates but these can be updated with country-specific information when available. The proportion of diarrhea deaths attributed to rotavirus is used in *LiST* as the *affected fraction*. Country level data with regard to rotavirus strains could also provide additional data to further refine the estimate of effectiveness of the vaccine or the pathogenicity of the specific strain of rotavirus circulating in a specific population, but these data are rarely available.

The variation in efficacy of the vaccine by region is important but additional data with regard to understanding what fraction of the observed variation is due to differences in strains versus host response will be critical to continue to improve upon current vaccine efficacy and the *LiST* effect size estimates. Some have speculated that vaccine efficacy might be influenced by such things as breastfeeding status, nutritional status, environmental enteropathy (that is, subclinical infections), or other environmental factors but additional research is needed to understand more fully how, and if, these factors may or may not be important [25].

*LiST* does not include default measures of herd immunity. *LiST,* as a model, is able to include the effect of herd immunity for any vaccine or intervention where herd immunity is important. At the present time, effect sizes for herd immunity have not been generated but experts are currently working on finalizing these estimates for rotavirus vaccine. When these data become available the overall effect of rotavirus vaccine in *LiST* will be changed to include herd immunity.

#### Impact of water, sanitation, and hygiene interventions for the prevention of diarrhea on diarrhea mortality

Water, sanitation, and hygiene (WASH) interventions include a wide spectrum of interventions, ranging from individual level behavior modification, such as handwashing promotion, to large-scale infrastructure interventions, such as the provision of piped water into the home. *LiST* includes the following interventions for the prevention of diarrhea: promotion of handwashing with soap; having access to an improved water source; a water connection in the home; improved sanitation source (that is, latrine/toilet); and the hygienic disposal of children’s stools. By nature, WASH interventions do not lend themselves well to blinded study designs and it is extremely difficult to randomize individuals in WASH studies; thus, there are no gold standard studies for which to determine effect sizes. Many studies are observational designs and in these studies there is often inadequate controlling for confounding [26]. In addition, many WASH studies were never designed to measure reductions in severe morbidity or mortality and WASH interventions are often presented in a package such that the effect of a group of interventions is being measured and thus attributing the effect of one intervention on any morbidity reduction is impossible.

Handwashing with soap is an important intervention for the prevention of diarrhea included in *LiST.* However, measuring this intervention is extremely difficult. Blinded studies cannot be done and non-blinded studies are subject to the placebo effect when asking caregivers about diarrhea incidence. By being part of a handwashing study, families may have adapted other more sanitary behaviors in the home and may also recall diarrhea less frequently, convinced that better habits have changed diarrhea incidence. Summary measures of water quality interventions are difficult to calculate because of the diversity of settings which directly influences the quality of the baseline water and the number of different kinds of interventions from home-based and home-made improved water solutions, such as filters, to commercial purification systems or tablets and the ideal scenario of piped clean drinking water. Waste disposal ideally occurs in an appropriately designed and maintained latrine or toilet, but these are still not accessible to many of the rural poor. Large randomized studies are rare due to the cost of building and maintaining latrines and toilets for large sample sizes and observational studies cannot fully control for clear differences in families that are able and willing to invest in a latrine versus those that are not able or chose not to.

Although the available literature does not provide ideal study designs and the overall quality of the data are very weak for some interventions, the consensus is that WASH interventions are important for the prevention of diarrhea [26]. These limitations should be considered when assessing the effect sizes included in *LiST* and might be further tailored when considering data from any one particular country. In the recent review by Cairncross *et al*. [26], the authors point out the limitations of the available data and encourage users of *LiST* to exercise caution with regard to the certainty of the size of the effect.

#### Impact of routine vitamin A supplementation on diarrhea mortality

Vitamin A supplementation every six months for the prevention of all-cause mortality is recommended for children six months to five years of age. In the recent review by Imdad *et al*., for use in *LiST*, the authors included both individual and cluster randomized trials [27]. Vitamin A supplementation has a direct effect on diarrhea mortality in *LiST* by reducing the mortality risk in vitamin A deficient children. Vitamin A reduces both the severity of disease (that is, the direct effect on mortality) and the incidence of disease (that is, the indirect effect on mortality) and both are included in *LiST*[13]. Most vitamin A studies were conducted more than 20 years ago in settings known to be vitamin A deficient. The proportion of children who are vitamin A deficient and, thus, who would benefit from vitamin A supplementation varies by region and is represented in *LiST* by regional specific affected fractions [13]. New data primarily generated from neonatal studies have suggested that there may be differences in effect size between Asia and Africa, but in a subgroup analysis of studies included in current estimates for children 6 to 59 months, there is not a statistically significant difference [27]. Coverage estimates need to reflect this twice yearly supplementation recommendation for valid use in *LiST.*

*LiST* does not currently include an estimate for vitamin A supplementation on diarrhea mortality associated with neonatal vitamin A supplementation. As new studies are conducted and the evidence base grows, this intervention will be re-considered for inclusion in *LiST*. At present there is not enough evidence to suggest a beneficial effect of vitamin A in this age group [27].

#### Impact of preventive zinc supplementation on diarrhea mortality

Routine zinc supplementation has the ability to reduce diarrhea morbidity and mortality especially among children who are zinc deficient [28]. While it is not yet a widely used intervention and not an intervention included in routine Demographic and Health Surveys (DHS) and Multiple Indicator Cluster Surveys (MICS), zinc supplementation for children one- to four-years-old is included in *LiST.* The effect sizes in *LiST* are based on a systematic review published as part of the *LiST* review supplements with later adjustments made by the *Lancet* Nutrition Series Working Group to include more tailored estimates of morbidity and mortality reduction based on the percent of the population at risk for zinc deficiency [28-30]. Routine zinc supplementation now operates in *LiST* in the same way as vitamin A, that is through an affected fraction, the percent of the population of children in the targeted age group at risk of deficiency. This improvement will be important and will ensure that the estimates of the effect on morbidity and mortality remain relevant as the background nutritional status of the population under five years of age continues to improve. Within *LiST* this remains an intervention that is usually included only in hypothetical scale-up models of lives that could be saved because very few places have widespread routine supplementation programs in place. Interventions, such as daily multiple micronutrient sprinkles containing zinc, may or may not have similar effects on diarrhea morbidity and mortality and, thus, cannot be interchanged with this intervention until the appropriate level of evidence is generated.

#### Impact of zinc supplementation for the treatment of diarrhea on diarrhea mortality

Zinc for the treatment of diarrhea has been recommended by WHO and UNICEF as an adjunct treatment for all episodes of diarrhea among children under five years of age since 2004 [3]. The randomized placebo controlled trials that generated the efficacy data which led to this recommendation were not designed or powered to detect differences in all-cause or cause-specific mortality; rather, these were treatment studies designed to measure the benefit of zinc on the duration and severity of the treated episode [31]. The systematic review that generated the evidence for the estimate currently included in *LiST* includes data from a literature review that included studies published from 1980 to 2009 [31]. A new review was conducted recently including studies published through 2012; this review also includes studies published in Chinese that had not previously been included in any review [32]. The results of this review will provide updated estimates for outcomes, such as the effect of zinc on diarrhea duration and severity, but will not change the *LiST* estimate because none of the new studies were effectiveness studies and they did not include the outcome of hospitalizations, which is what current *LiST* estimates are based on.

To date, there has been one study published that found an observed 66% (95% confidence interval (CI) −37, 96%) reduction in diarrhea specific mortality, but even this large community based study was not powered to detect cause-specific reduction in mortality [33]. Four studies report reductions in all cause mortality. Although the results of the pooled analysis produced a statistically significant reduction (46%, 95% CI: 12% to 68%) [31], it is important to note that these studies were not designed to measure the effect of zinc on mortality [33-36]. There were also very few deaths in these four studies. The rules of the *LiST* tool dictate that when there are fewer than 50 events for a given outcome, the effect size of the intervention on a less severe outcome should also be considered and the smallest effect size (that is, the most conservative estimate) used [37]. In the case of zinc supplementation, the reduction in hospitalizations was 23% and there were at least 50 hospitalizations overall among the studies that measured hospitalization so this estimate was then adapted by *LiST* for the effect size of zinc [31].

In the case of zinc, the 23% reduction in hospitalizations can be argued to be a very conservative effect size estimate as it is based on community-based intervention studies and measured reductions in hospitalizations in the community; however, in these studies coverage did not reach 100%. In Bangladesh, coverage reached more than 80% [33] and in India coverage reached nearly 60% [38]. It is important to note that in these two studies, the recommended duration of treatment was 14 days; the WHO/UNICEF recommendation is 10 to 14 days of zinc. In India, 61.7% of children received the full 14 days of treatment [38]. In Bangladesh the mean course of treatment was seven days [33]. Both studies also promoted ORS and observed increases in ORS use rates in the villages that received zinc; thus, it is difficult to isolate the effect of zinc alone in these treatment studies. The 23% reduction in mortality that is used in *LiST* for the effectiveness of zinc treatment is consistent with the observed 25% reduction in prolonged diarrhea based on efficacy studies which were all individually randomized, unlike the cluster randomization in the effectiveness studies [31]. Some studies have shown a reduction in diarrhea incidence in the two to three months following the treated episode, but this is not included in *LiST*[39].

#### Impact of oral rehydration salts for the prevention and treatment of dehydration on diarrhea mortality

ORS has been the cornerstone of diarrhea treatment since the 1970s, preventing and treating dehydration. In 2004, WHO updated the recommendation to a new lower osmolarity version of ORS [40,41]. Low osmolarity ORS prevents and treats dehydration more effectively by decreasing the incidence of vomiting, lowering stool output, and decreasing the likelihood of needing intravenous therapy (IV) [40]. The early studies demonstrating the efficacy of ORS were controlled trials assessing ORS versus IV therapy as it would have been unethical to include a placebo for the treatment of dehydration [42]. After efficacy was proved, researchers moved to community-based intervention studies introducing ORS also as a preventive tool for dehydration [42]. These studies often compared more aggressive delivery strategies with routine delivery and measured differences in coverage to determine the added effect of ORS on mortality.

Past Cochrane reviews included the outcome of treatment failure for original ORS, low osmolarity ORS, and a comparison of rice-based versus glucose-based ORS [40,43,44]. All Cochrane reviews included studies conducted in hospital and clinical settings. ORS, as an intervention in *LiST,* is a community-based intervention for the prevention of diarrhea mortality in the community and in clinical settings. Munos *et al*. conducted a new systematic review including many diverse study designs (quasi-experimental, pre/post and observational) and studies conducted in the community as well as in hospital/clinical settings [42]. For the three large community-based studies that assessed diarrhea mortality, the reported reduction in mortality is associated with an achieved coverage rate [45-47]. For the purposes of calculating the potential reduction in diarrhea mortality, the authors assumed a linear trend between the relative reduction in mortality and achieved coverage, such that if ORS reduces diarrhea mortality by 69% at 74% coverage, then ORS would reduce mortality by 93% at 100% coverage [42]. It is important to note that community-based studies have some level of coverage of ORS in control communities, which is not taken into account, and thus this estimate is a conservative estimate and may overestimate the true coverage needed to reach the estimated mortality reduction.

*LiST* does not include other strategies for the prevention of dehydration, such as the provision of recommended home fluids (RHF). Providing fluids, other than ORS, for the prevention of dehydration became popular in the 1980s with a movement for home-based management without having to purchase any outside therapy. Sugar-salt-solution, the closest home-made solution to ORS, was tested in hospitals and found efficacious [42]. That is, if prepared in a hospital setting it could be used successfully but it did not go through testing in community settings, the very setting where it is intended for use. Other fluids were meant for the early prevention of dehydration in the home and were never meant for the treatment of dehydration and never formally tested.

#### Impact of antibiotics for the treatment of dysentery on diarrhea mortality

The selective use of antibiotics for the treatment of dysentery is a key component of diarrhea management [3]. In low- and middle-income countries, dysentery and, more specifically, dysentery deaths are typically caused by *Shigella*[21,48,49]. Beyond treating the episode of dysentery, antibiotic treatment is important in decreasing the bacterial load in excrement and then decreasing the risk of fecal-oral transmission in the household and community [50]. *LiST* includes estimates of effectiveness of the WHO-recommended antibiotics (that is, ciprofloxacin, ceftriaxone and pivmecillinam) on dysentery. The *LiST* estimate of 99% reflects the rate at which clinical signs and symptoms of *Shigella* were eliminated [51]. This estimate is in line with estimates of bacteriologic failure rates of 99% to 100% [51,52]. The *LiST* estimate operates through an affected fraction which, in this case, is 3.9% (that is, the percent of diarrhea deaths attributable to *Shigella*) [21]. Thus, in *LiST* if the coverage of antibiotics for the treatment of dysentery were to be 100%, 3.86% of diarrhea deaths (that is, 99% of 3.9%) would be prevented.

Antibiotics are widely and inappropriately used for the treatment of routine acute diarrhea in many countries. Anti-diarrheals are also common. The coverage of antibiotics is difficult to trace in coverage surveys because differentiating between antibiotics and anti-diarrheals is difficult. Where the caregiver is no longer in possession of the bottle or packaging and in countries where numerous antibiotics and anti-diarrheals are available, the caregiver is often unable to remember or correctly identify which drug was given to the child. For these reasons, accurately measuring coverage of antibiotics is challenging [53].

### Improving data inputs will improve *LiST* outputs

#### Diarrhea etiology

Childhood infectious diarrhea is typically caused by viruses, bacteria and parasites. The etiology of acute diarrhea has been widely studied among incident cases yet, in low- and middle-income countries, the causes of most cases of acute diarrhea are never investigated as part of routine treatment protocols. Estimates of the pathogen specific causes of diarrhea deaths are extrapolated from hospitalized cases because global data for pathogen specific cause of death do not exist for low- and middle-income countries [21]. For these reasons, in *LiST* very few diarrhea interventions are diarrhea pathogen-specific. *LiST* does incorporate an estimate for the proportion of diarrhea deaths attributable to rotavirus; the rotavirus vaccine prevents deaths from within this affected fraction. *LiST* also incorporates an estimate for the fraction of deaths attributable to dysentery, clinically recognized as blood in the stool. The scale up of antibiotic treatment for dysentery prevents deaths from within this affected fraction of all diarrhea deaths. As new interventions are made available, for instance, a program to include cholera vaccine, *LiST* is able to incorporate etiologic specific mortality by including an estimate of the proportion of deaths attributable to a specific etiology. Although all etiologies are not currently included in *LiST,* recent estimates for the most important causes of diarrhea are available and could be included as needed [21].

### New interventions

*LiST* also has a feature that allows users to add new interventions into the model. To do this, users must specify the links between the new interventions and outcomes (risk factors or causes of mortality) as well as the efficacy of the intervention on the outcomes to which it has been linked. Also for diarrhea, *LiST* has two additional vaccines (called vaccine B and vaccine C) included as ‘dummy vaccines’ for testing the possible impact of new vaccines. Users can link one of these ‘dummy vaccines’ to etiologically specific causes of diarrhea mortality and morbidity. In the standard version of *LiST,* the links and efficacy values for these two vaccines are left blank, but the user can define the linkages and efficacy values. These extra vaccines were added to the model as there has been much work in the development of vaccines for diarrhea for enterotoxigenic *Escherichia coli* (ETEC) and *Shigella*. This feature also allows a user to estimate the impact of cholera vaccines if that vaccine is used in a country.

### Accuracy of *LiST* estimates and possible limitations to the model

There has been extensive work comparing the estimates that come from *LiST* to measured changes in intervention coverage and mortality. Several studies have compared measured changes in mortality to *LiST* estimates of mortality change looking at different sets of interventions in different countries. For example, one study compared *LiST* estimates to measured reduction in neonatal mortality in community trials in South Asia [54]. Another study looked at community trials that focused on the scale up and use of insecticide treated nets (ITNs) in sub-Saharan Africa [55]. A third compared measured and estimated mortality for a community trial in Mozambique [56]. In all of these studies there was close agreement between the estimates of mortality from *LiST* based on coverage changes and the measured reductions in mortality. Additional studies doing comparisons of *LiST* have been published in the *LiST* journal supplements [57-59].

While the comparison work has generally found good agreement between the *LiST* estimates and measured mortality reduction, there are issues or circumstance that could make the *LiST* estimates less accurate or the model less robust. These issues generally fall into two categories; one related to data availability and the second to the general structure and assumptions of the model. These issues will be discussed below.

One of the major issues with regard to accuracy of the *LiST* model is the availability of data on coverage of interventions. While most low- and middle-income countries do have periodic surveys that measure coverage of key interventions for maternal and child health (as well as for risk factors such as breastfeeding patterns, stunting and wasting) these surveys often occur only every three to five years. This means that for many countries when we build a baseline, the coverage data are not current. A second issue related to coverage is that while survey questions yield the base data we have on coverage of interventions for most low- and middle-income countries, the surveys themselves do not necessarily provide highly accurate measures of coverage of many interventions. In May 2013, *PLoS Medicine* published a series of papers that evaluated how well household surveys, such as MICS and DHS, measured coverage of selected key interventions for maternal and child health [60]. These studies found that surveys often do not provide highly accurate estimates of coverage, especially for treatment interventions, such as antibiotics for pneumonia and the treatment of diarrhea [53,61]. Finally, there are interventions used in *LiST* where we have no reliable source of coverage information from the major household surveys. For example, for many of the interventions during antennal care and child birth, surveys only capture the number of antenatal visits, or locations and type of service provided for child birth, not a measure of the sub-component services (for example, detection and treatment of syphilis or diabetes for pregnant women; availability of core services that comprise basic or emergency obstetric care during child birth). Instead in *LiST* we used data from a small set of studies to estimate coverage change of the actual interventions as they relate to the indicators measured in surveys (for example, four antenatal visits, skilled birth attendant, and institutional delivery). For diarrhea interventions, the intervention for which we have the poorest measures of coverage is hand washing with soap, as reported behaviors are often unreliable and direct observation is very difficult; thus, this measure is particularly problematic [26].

In terms of limitations or restrictions to the wide-spread appropriate use of *LiST* in modeling mortality reduction there are two primary issues that have been raised by reviewers of papers using *LiST* to model mortality reductions. These are the inability of *LiST* to capture secondary effects of an intervention on overall mortality (as *LiST* models effects of interventions on cause-specific mortality) and the assumption that coverage measures of interventions are independent. We will discuss these issues separately below.

The idea of secondary effects of interventions not captured in *LiST* is primarily based on the idea that if one reduces the incidence and mortality of one disease due to an intervention the reduction in one disease will result in the child being healthier and less vulnerable to the exposure of a second disease. For example with diarrhea, people have argued that as we have reduced the incidence of measles due to the high coverage of measles vaccine, measles as a diarrhea risk factor has been drastically reduced and, thus, children are less likely to develop diarrhea and for those who have diarrhea less likely to die [62]. Currently, *LiST* does not include estimates for secondary effects. For example, a measles vaccine reduces measles incidence and mortality, but has no effect on diarrhea deaths. Of course, conceptually this could be added to the model quite easily, as interventions can have effects on more than one cause of death. However, to date most intervention reviews have not found a significant effect of the secondary causes of mortality. One of the reasons that some argue that *LiST* is missing the significant impact of interventions via secondary effects is that trials that have looked at the impact of an intervention on all-cause mortality often find larger effects on all-cause mortality than would be expected if there were no secondary effects. For example, one of the early insecticide-treated bed net trials found a more than 50% reduction in all-cause mortality among children when the estimated proportions of deaths due to malaria were estimated to be around 40%. The same comparisons have been used to also argue that *LiST* use of efficacy values linked to cause-specific mortality has under-estimated the overall impact of interventions, such as vaccines and vitamin A supplementation [63]. However, when data have been available to compare *LiST* estimates that do not include these secondary effects to measured all-cause mortality, there has been little evidence that *LiST* systematically under-estimates the effects. For more details on this see the analyses done by Larson *et al*. [55].

Another possible issue with the structure of *LiST* is that it assumes that coverage estimates of interventions, when scaled up, are independent. Basically, this assumes that there is not a large section of the population that has little or no chance of getting any interventions. Theoretically, if coverage of one intervention was strongly associated with coverage of others (and clearly there is some association) then *LiST* could produce biased estimates of the impact of scaling up coverage at the population level. Victora and colleagues addressed the theoretical and actual impact of equity of coverage [64]. In this simulation the authors showed that there was a potential and sizable bias with *LiST* estimates if coverage of interventions were strongly correlated. However, the authors also estimated the observed correlations between interventions in a set of low- and middle-income countries and found that the degree of correlation of intervention coverage was not sufficient to lead to a sizable bias in *LiST* estimates of the impact of interventions.

### Using *LiST* as a tool within a comprehensive program evaluation

Within the scope of program evaluation, donors or policy makers are often concerned about whether or not a program has had an impact, that is, has there been a measurable change on mortality or on nutritional status and can this impact, if any, be attributable to a specific program. *LiST* relies on accurate measurements of coverage and with these has been shown to produce precise measures of cause-specific mortality reduction [54,65]. However, measuring coverage and modeling its impact on mortality is only one key component for a complete program evaluation. Bryce *et al*. have published a common evaluation framework that clearly lays out the many components of a complete program evaluation highlighting the leanings that are needed at multiple stages [4]. A complete evaluation is able to capture data about inputs, processes and outputs, not just outcomes and impact. *LiST* does not have the capacity for describing why coverage of a certain intervention increased or decreased beyond the indicators that are measured routinely in surveys. Nor does it have the ability to give guidance on ‘how’ coverage of an intervention could be changed.

## Summary

*LiST* is an excellent tool that has been used for many years and has undergone numerous improvements over time; continued efforts to further refine inputs will continue to improve the model for years to come. *LiST* has been tested many times to ensure the modeled mortality estimates that it produces are valid and reproducible [54,65]. The strength of *LiST* is dependent upon the strength of the input data. *LiST* continues to update child cause of death and overall mortality rates and includes the most recent DHS and MICS coverage data for all included child survival interventions*.* For time periods that do not correspond to preset coverage surveys, *LiST* allows a user to input different coverage estimates and, thus, *LiST* can be used to model anticipated reductions in child mortality if a given package of interventions were to be rolled out for a specified project period. Ideally, every coverage survey rolled out as part of program evaluation would measure the coverage of all key child survival interventions over the time period of interest. With these data, *LiST* is able to account for changes in overall mortality or cause of death structure and more precisely attribute any changes in impact to the interventions in question. Where limited coverage data are available, the user has the ability to look at the impact of an isolated group of interventions by keeping coverage estimates for other interventions constant or can factor in past time trends to include the influence of time trends of other interventions but both of these methods introduce tremendous bias. *LiST* is an effective tool for modeling diarrhea mortality and can be a useful alternative to large and expensive mortality impact studies. *LiST* will continue to evolve as additional data are collected from trials and studies to better define coverage*.* Child mortality rates are rapidly declining and, thus, the importance of tools such as *LiST* is becoming increasingly important. Predicting the impact of interventions or comparing the impact of more than one intervention without having to wait for the results of large and expensive mortality studies is critical to keep programs focused and results oriented for continued reductions in diarrhea and all-cause mortality among children under five years of age.

## Abbreviations

CI: confidence interval; DHS: Demographic and Health Surveys; LiST: Lives Saved Tool; MICS: Multiple Indicator Cluster Survey; ORS: oral rehydration salts; WASH: water, sanitation and hygiene.

## Competing interests

The authors declare they have no competing interests.

## Authors’ contributions

CLFW and NW conceptualized the paper and jointly wrote the manuscript. Both authors read and approved the final manuscript.

## Supplementary Material

Additional file 1 Web Appendix. Click here for file
